# Induction Time of Wetting Films Between Air Bubbles and Hydrophobic Particles in the Presence of Dodecyl Amine Hydrochloride: First Principles Model Analysis and Experimental Validation

**DOI:** 10.3390/molecules30030695

**Published:** 2025-02-05

**Authors:** Boris Albijanic, Anh V. Nguyen, Orhan Ozdemir, Arturo A. García-Figueroa

**Affiliations:** 1 WA School of Mines: Minerals, Energy and Chemical Engineering, Curtin University, Kalgoorlie, WA 6430, Australia; 2 School of Chemical Engineering, The University of Queensland, St. Lucia, QLD 4072, Australia; 3 Department of Mineral Processing Engineering, Faculty of Mines, Istanbul Technical University, 34485 Istanbul, Türkiye; orhanozdemir@itu.edu.tr

**Keywords:** bubble–particle attachment mechanism, induction time, intermolecular forces, surface rheology

## Abstract

An attachment of a particle on a bubble is a very complex process due to the surface chemistry of bubbles and particles, and hence it is difficult to describe the bubble–particle attachment mechanism from first principles. This paper focuses on better understanding of the bubble–particle attachment mechanism by predicting induction time from first principles for the glass beads–dodecyl amine hydrochloride (DAH) system. The induction time for the bubble–particle attachment was determined using an optically based attachment timer. The zeta potentials of bubbles and glass particles were measured by the microelectrophoresis method. The contact angle between a bubble and a particle was obtained using atomic force microscopy. In these calculations, the overall disjoining pressure and the overall energy of bubble–particle interactions comprised a sum of the DLVO and non-DLVO contributions. The overall energy of interactions was used to determine the critical film thickness, while the overall disjoining pressure was employed to estimate the wetting film drainage time using the theoretical models. By comparing experimental data and theoretical models for drainage of wetting films, it was found that the attachment of glass particles to air bubbles in the presence of DAH is accelerated due to the mobility of the air–water interface (of the wetting films), which has incorrectly been assumed as rigid (fully immobile) in the classical Stefan–Reynolds theory.

## 1. Introduction

Understanding bubble–particle interactions is crucial, particularly in flotation processes, which are extensively employed for recovering valuable minerals, deinking wastepaper, treating water, and extracting oil from tar sands [1]. While these interactions involve collision, attachment, and detachment, the attachment between bubbles and particles is the most critical for achieving effective flotation-based separation. However, it remains the least understood aspect due to the complex surface chemistry of particles and air bubbles [2].

Bubble–particle attachment interactions consist of three subprocesses: (1) the thinning of wetting films between a bubble and a particle, (2) the rupture of the wetting film, and (3) the expansion and relaxation of the gas–liquid–solid contact lines [3]. The time required for the first two subprocesses is referred to as the induction time, while the total duration encompassing all three subprocesses is termed the attachment time [4]. The attachment time is measured using a device developed by Glembockij [5], which employs a captive bubble brought into contact with a particle bed for controlled time intervals. The attachment time corresponds to the contact duration at which a predetermined percentage of observations (e.g., 50%) results in bubble–particle attachment. The attachment time includes the wetting film drainage time, rupture time, and gas–liquid–solid line expansion time. However, measurements obtained with the Glembockij device closely approximate the wetting film drainage time, or induction time. Experimental confirmation of this approximation was provided by Wang et al. 2005, who used a high-speed camera to demonstrate the similarity between attachment time and induction time [6].

Induction time measurements are commonly used to investigate the mechanism of bubble–particle attachment. A short induction time (1–20 ms) indicates strong surface hydrophobicity, whereas a long induction time suggests weak surface hydrophobicity. Induction time has proven to be highly sensitive to the surface chemistry and physical properties of particles. For instance, the measured induction time varies with particle composition, as differences in composition can result in varying levels of hydrophobicity [4,7,8,9]. While induction time can be effectively measured experimentally, developing accurate mathematical models for induction time remains challenging due to the complexity of wetting film drainage between bubbles and particles.

Mathematical models for the drainage rate of wetting films can be categorized into two groups [4]: those designed for films with a large radius and those for films with a small radius. Models for large-radius film drainage are applicable when the wetting film forms between a bubble and a flat solid surface. In such cases, the film exhibits a dimpled profile with a non-uniform thickness, as the drainage rate at the edges of the film is faster than at its center. A comprehensive review of these models is provided in Ref. [10]. Models developed for the drainage of wetting films with a small radius are essential for mathematically describing bubble–particle attachment in flotation separation. In this case, the drainage process does not produce dimpled profiles; instead, the film thickness remains uniform across its radius [3]. These models are derived from the Navier–Stokes and continuity equations, using the lubrication approximation, which assumes the film thickness is much smaller than its other dimensions [3].

Two models describe the drainage of wetting films between bubbles and particles: the Taylor equation and the Stefan–Reynolds equation [7]. The Taylor equation applies to wetting film drainage during sliding interactions, where colloidal forces are negligible. Conversely, the Stefan–Reynolds equation is used for film drainage between plane parallel, immobile surfaces during collision interactions. Since the Glembockij device measures induction time during collision interactions, the Stefan–Reynolds equation is typically employed. This equation has been experimentally validated for wetting films stabilized by repulsive DLVO (Derjaguin–Landau–Verwey–Overbeek) forces, which arise from van der Waals and electrical double-layer (EDL) interactions [11]. These validations were conducted on hydrophilic glass surfaces. However, when studying thinning on hydrophobic glass surfaces, the induction time calculated using the Stefan–Reynolds equation, considering both repulsive DLVO forces and attractive non-DLVO forces (hydrophobic interactions), was significantly shorter than the experimentally measured values. This discrepancy may be due to the absence of long-range hydrophobic forces. Additionally, deviations from the Stefan–Reynolds equation have been attributed to surface mobility effects [12,13,14,15,16]. To address this, Tsekov et al. 1998 [16] extended the classical Stefan–Reynolds equation by incorporating the influence of surface rheology from adsorbed surfactants on wetting film thinning. However, this extended model has not yet been experimentally validated. It is also important to note that models describing wetting film drainage between bubbles and particles generally neglect the deformation of the gas–liquid interface during thinning. Atomic force microscopy (AFM) studies [17,18] have confirmed this limitation. Nonetheless, these models serve as a useful first approximation for estimating induction time in flotation.

Despite the various models proposed to mathematically describe wetting film drainage, accurately predicting the induction time for films between air bubbles and hydrophobic surfaces remains a significant challenge. This study aims to address this issue by predicting the induction time from first principles to enhance our understanding of the bubble–particle attachment mechanism in a glass beads–dodecyl amine hydrochloride (DAH) system. The extended DLVO theory was applied to calculate the critical film thickness and disjoining pressure, while the classical Stefan–Reynolds theory and the model proposed by Tsekov et al. [16] were used to describe the thinning of wetting films. Comparing experimental results with predicted induction times will provide valuable insights into bubble–particle attachment interactions.

## 2. Theory

### 2.1. Theoretical Models for Wetting Film Drainage

The wetting film drainage theory for plane parallel immobile surfaces can be described using the Stefan–Reynolds equation [3]:

(1) $$\frac{dh}{dt}=-\frac{2h^{3}}{3\mu R^{2}}\left(P_{\sigma }-\Pi \right)$$

where *h* is the thickness of the wetting film, *μ* is the bulk liquid viscosity, *P_σ_* is the capillary pressure as a function of the surface tension *σ* and particle size *R_p_* (180 µm) (*P_σ_* = *2σ*/*R p*), *Π* represents the disjoining pressure, and *R* is the film radius, which can be estimated applying the empirical model [19] as

(2) $$R=\frac{\pi R_{p}\left(656.9-87.4\ln R_{p}\right){\left[V_{rel}t_{c}\right]}^{0.62}}{180}$$

where *R_p_* is measured in µm, *V_rel_* and *t_c_* are the bubble–particle approach speed (0.3 cm/s) measured in (cm/s) and the collision contact time measured in (s), respectively. It should be noted that the accurate prediction of film radius is still very difficult. For example, Atard and Miklavcic (2001) [17] used first principle modelling to develop the model for predicting a film radius $\sqrt{2R_{p}k^{-1}}$ (*k*^−1^ is the Debye length −96 nm for deionised water). However, the calculated film radius is then 6 µm, which is 18 times lower than the experimental value obtained by Schulze [19]. For that reason, in this paper, Equation (2) will be applied to calculate film radius.

The departure from the Stefan–Reynolds equation occurs because of the surface mobility [12,13,14,15,16]. Therefore, Tsekov et al. 1998 [16] developed a new theory for the drainage of wetting films by considering the mobility of the air–water interface. The Tsekov drainage model can effectively be described by Equation (1), with the viscosity (*µ*) being replaced by an effective viscosity, *μ_eff_*, which can be described as:

(3) $$\mu_{eff}=\mu \frac{Ma+Na+Ap+MaNa+ApNa/2}{12+4Ma+4Na+MaNa}$$

In Equation (3), $Ma=ahE/\mu (D_{s}a+Dh)$, Na=hβ/μ, and $Ap=-ah^{2}\Gamma (\partial \Pi /\partial \Gamma )/\mu \left(D_{s}a+Dh\right)$ represent the Marangoni, Navier, adsorption–pressure numbers, respectively, *a* is the adsorption length, *a* = *dΓ*/*dC*, and *E* = −*dσ*/*dlnΓ* is the Gibbs elasticity, where *σ*, *C,* and *Γ* describe the surface tension, surfactant concentration in the bulk solution, and the surfactant adsorption density at equilibrium, respectively. *D_s_* and *D* are the diffusion coefficients of the surfactant on the surface and in the bulk solution, respectively. *β* is the slip coefficient on the solid/wetting film interface. For the experimental systems in this work, the Navier and adsorption pressure numbers were negligibly small, and thus the effective viscosity can be simplified as

(4) $$\mu_{eff}=\mu \frac{Ma}{12+4Ma}$$

### 2.2. Intermolecular Interaction and the Surface Forces

The disjoining pressure is very important to model the wetting film drainage time. The theoretical description of the disjoining pressure depends on molecular interactions between a bubble and a particle [3].

The van der Waals (vdW) interactions involve the Keesom (interaction between two randomly oriented dipoles), the Debye (interaction between a permanent dipole and an induced dipole), and the London (interaction between a fluctuating dipole and an induced dipole) contributions [3]. Although the Lifshitz approach is used to determine the vdW interactions accurately, this approach requires the information about the relative permittivity materials under the entire frequency range, which is not always available for practical applications [3,20]. Therefore, the simple Hamaker approach is often suitably used in the modelling of the bubble–particle interactions. The Hamaker theory gives

(5) $$\Pi_{vdw}=-\frac{A_{123}}{6\pi h^{3}}$$

where *A*_123_ is the Hamaker constant for the interaction between a bubble and a particle immersed in the same solution. It is important to note that for bubble–particle interactions, the Hamaker constant is always negative, and thus the vdW interactions are repulsive [3]. The magnitude of the Hamaker constant is of the order of 1 × 10^−20^ J. Therefore, the van der Waals pressure is significantly smaller than the EDL pressure, unless the film thickness is smaller than 5 nm. Since the wetting films are ruptured at a thickness significantly larger than 5 nm, the vdW disjoining pressure will be neglected in our calculation.

The electrostatic double layer (EDL) interactions occur due to the overlapping of electrical double layers of bubble and particle [3]. The Poisson–Boltzmann equation is used to determine the EDL disjoining pressure. Because this equation is highly non-linear, the Debye–Hückel approximation is employed to calculate the EDL disjoining pressure. When the model for disjoining pressure is known, the Derjaguin approximation is applied to determine the energy of the EDL interactions. To obtain models for the disjoining pressure or the energy of EDL interactions, it is also very important to know the type of the surface charging mechanisms. The two surface charging mechanisms usually considered are the constant surface potential case or the constant surface charge case [3]. The expressions for disjoining pressure and the energy of interactions when both bubble and particle have constant surface potentials are given as:

(6) $$\Pi_{edl}^{\psi }=\frac{\varepsilon \varepsilon_{0}k^{2}}{2}\frac{2\psi_{1}\psi_{2}\cosh\left(kh\right)-\psi_{1}^{2}-\psi_{2}^{2}}{\sin^{2}\left(kh\right)}$$

(7) $$E_{edl}^{\psi }=\frac{\varepsilon \varepsilon_{0}\pi R_{b}R_{p}}{R_{b}+R_{p}}\left[4\psi_{1}\psi_{2}a\tanh\left(e^{-kh}\right)+(\psi_{1}^{2}+\psi_{2}^{2})\ln\left(1-e^{-2kh}\right)\right]$$

In the case when both bubble and particle have constant surface charge, the following models are obtained:

(8) $$\Pi_{edl}^{\psi }=\frac{\varepsilon \varepsilon_{0}k^{2}}{2}\frac{2\psi_{1\infty }\psi_{2\infty }\cosh\left(kh\right)-\psi_{1\infty }^{2}-\psi_{2\infty }^{2}}{\sin^{2}\left(kh\right)}$$

(9) $$E_{edl}^{\psi }=\frac{\varepsilon \varepsilon_{0}\pi R_{b}R_{p}}{R_{b}+R_{p}}\left[4\psi_{1\infty }\psi_{2\infty }a\tanh\left(e^{-kh}\right)+(\psi_{1\infty }^{2}+\psi_{2\infty }^{2})\ln\left(1-e^{-2kh}\right)\right]$$

where *R_b_* (1.5 mm) and *R_p_* (181 μm) are radius of a bubble and a particle, respectively; *ψ*_1_ and *ψ*_2_ represent the surface potentials for a particle and a bubble, while the shortest distance between them is *h*; *ε*_0_ is the vacuum permittivity (8.854 × 10^−12^ C^2^J^−1^m^−1^); and *ε* is the dielectric constant (80 for water).

Apart from the vdW and the electrostatic double layer interactions (the DLVO forces), the hydrophobic interactions (the non-DLVO forces) have also been identified as an important interaction in the drainage of wetting films between a bubble and a particle [2,3]. The existence of hydrophobic interactions has been confirmed using the surface force apparatus [21] or atomic force microscopy [22,23,24,25]. Namely, the hydrophobic force is determined as a difference between the measured force and the DLVO forces. The origin of this force has not yet been established, although different mechanisms have been proposed to explain it [3]. The empirical models are typically employed to predict the hydrophobic disjoining pressure, such as a single or a double exponential function [3]:

(10) $$\Pi_{hyd}=K\exp\left(-\frac{h}{\lambda }\right)$$

(11) $$\Pi_{hyd}=K\exp\left(-\frac{h}{\lambda }\right)+K^{*}\exp\left(-\frac{h}{\lambda^{*}}\right)$$

where *K* and K* are the pressure constants, and *λ* and λ* represent the decay lengths.

Tsekov and Schulze [26] proposed another theoretical model for the hydrophobic disjoining pressure, which includes the contribution of adsorption of surfactant on the solid surface:

(12) $$\Pi_{hyd}=\frac{∆E_{1}}{a_{1}}\exp\left(-\frac{h}{a_{1}}\right)+\frac{∆E_{2}}{a_{2}}\exp\left(-\frac{h}{a_{2}}\right)$$

where Δ*E* represents the difference between the Gibbs elasticity of a liquid–solid interface and that of a gas–solid interface, and the subscript describes the two film surfaces.

However, in this paper, because the data for this hydrophobic constant (*K*_123_) are available in the literature [2], the disjoining pressure will be determined using the power function:

(13) $$\Pi_{hyd}=-\frac{K_{123}}{6\pi h^{3}}$$

and then the energy of hydrophobic interactions is given as:

(14) $$E_{hyd}=-\frac{R_{b}R_{p}K_{123}}{6\left(R_{b}+R_{p}\right)h}$$

In bubble–particle interactions, the overall disjoining pressure and the overall energy of interactions comprise a sum of the vdW, the electrostatic double layer, and the hydrophobic contributions. As mentioned in this section, the vdW interactions will be neglected in all calculations. The overall energy of interactions will be used to predict the critical film thickness, while the overall disjoining pressure will be employed to estimate the wetting film drainage time.

## 3. Results and Discussion

**Figure 1:**
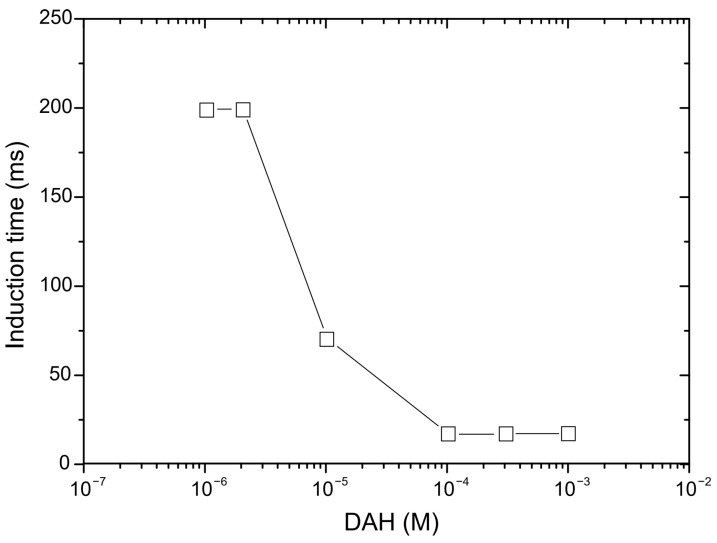
Induction time of glass particles on bubble as a function of DAH concentration.

The results for the induction time measurements of the glass particles are shown in Figure 1. As can be observed in Figure 1, the induction time of the particles sharply decreased with respect to the DAH concentration. The reason for this is due to the adsorption of DAH molecules on glass surfaces, in which hydrophilic glass particles converted to hydrophobic ones. At low concentration of DAH, the polar parts of RNH^3+^ ions adsorb at the particle surface, whereas the non-polar parts (hydrocarbon chain) orient toward the water phase. In the air–water interface, the polar parts orientate on the water side, while the non-polar parts orient to the air side. As the DAH concentration increases, both surfaces are covered by non-polar hydrocarbon chains, which drastically reduces EDL forces, decreasing inductions times [27].

**Figure 2:**
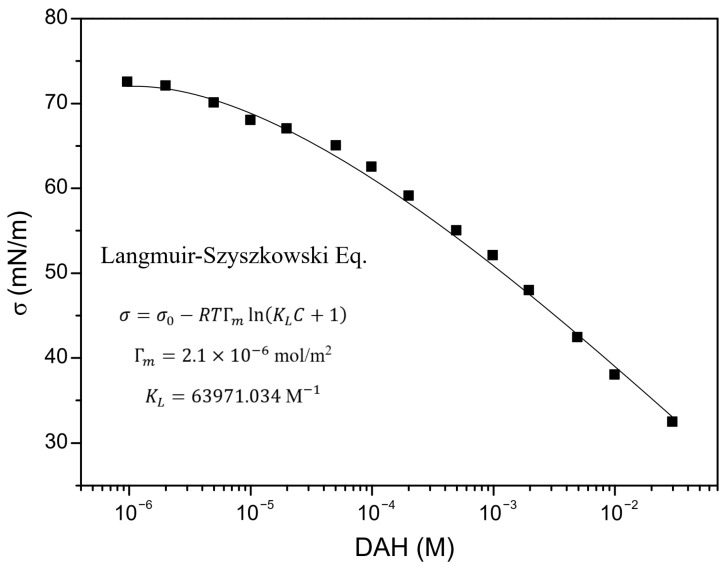
Surface tension of DAH. Adapted from Alexandrova et al. [28].

**Figure 3:**
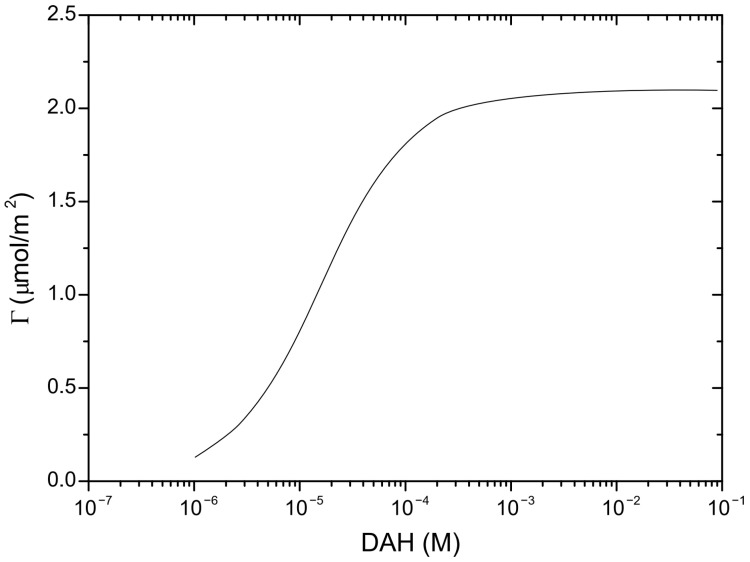
Surface excess at the air–water interface as a function of DAH concentration.

**Figure 4:**
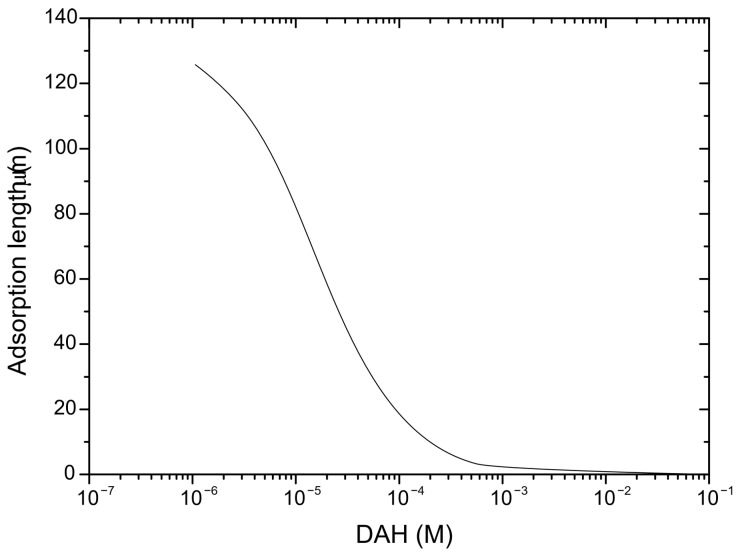
Adsorption length (*a* = *dΓ*/*dC*) using the data shown in Figure 3 and Figure 4.

**Figure 5:**
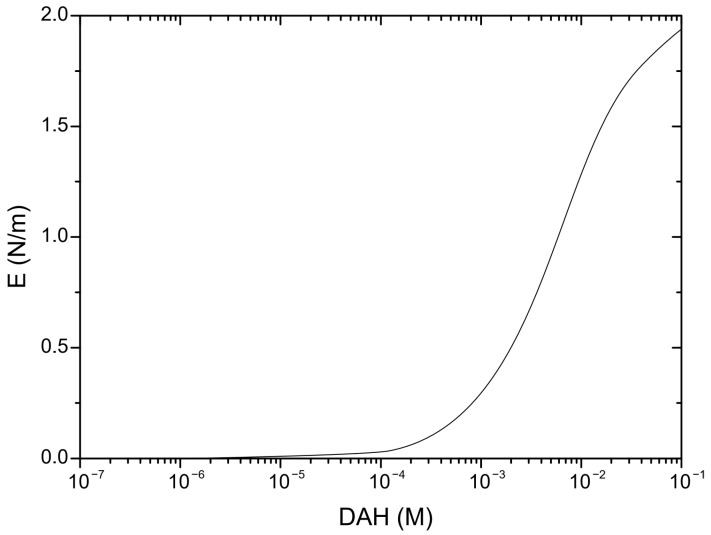
Gibbs elasticity (*E* = −*∂σ*/*∂lnΓ*) using the data shown in Figure 3 and Figure 4.

In order to predict induction time, it is important to determine the mobility of wetting liquid film surfaces, as well as critical film thickness (defined as the film thickness at which the wetting film ruptures spontaneously). The mobility of the wetting film surface was determined by applying the following procedure. The experimental results of surface tension for DAH solutions as determined by Alexandrova et al. [28] are shown in Figure 2. The parameters of surfactant adsorption (*Γ_m_* and *K_L_*) were obtained by minimizing the sum of the squares of the deviations of these measurements from the values predicted by the Langmuir–Szyszkowski model, as seen in Figure 2. Once parameters of adsorption are determined, the adsorption density of DAH at the air–water interface was calculated using the Langmuir isotherms. The Langmuir–Szyszkowski model (Figure 2) and the Langmuir isotherms (Figure 3) were employed to compute the Gibbs elasticity (*E* = −*∂σ*/*∂lnΓ*) and the adsorption length (*a* = *dΓ*/*dC*), shown in Figure 4 and Figure 5. Then, the Ma number was calculated (*Ma* = *ahE*/*µ(D_s_a* + *Dh)*), at which the surface diffusion coefficient for surfactant and diffusion coefficient of surfactant in the bulk solutions were estimated as 4 × 10^−9^ and 4 × 10^−10^ m^2^/s [29], respectively. Next, Equation (1) together with Equation (4) were applied to investigate the mobility of the wetting film surface. The results indicated that the relative viscosity (i.e., the ratio if the effective to bulk viscosity) is 0.25. Therefore, in this case, the Stefan–Reynolds lubrication approximation with slip boundary conditions can be used to describe the drainage of wetting films [3]. In other words, by applying the theory proposed by Tsekov et al. [16], it was found that one film surface was fully mobile while the other film surface was fully immobile. It should be noted that, in this case, the drainage of liquid film is four times faster than the drainage described by the classical Stefan–Reynolds equation.

**Figure 6:**
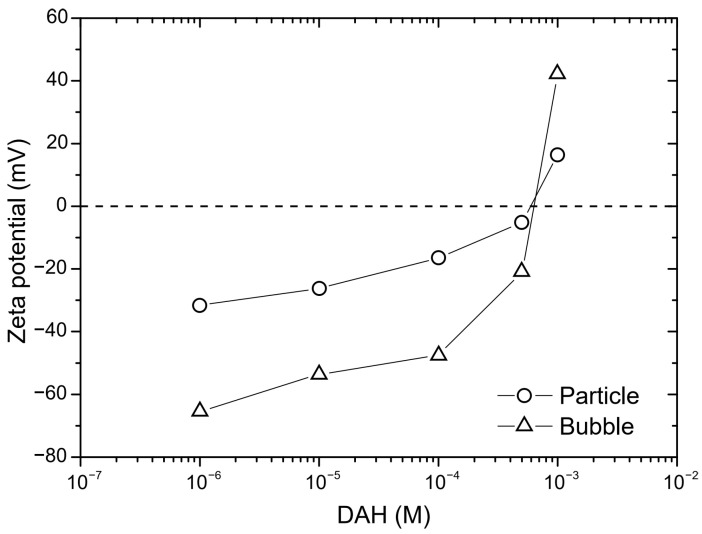
Zeta potentials of glass particles and air bubbles in the presence of DAH.

**Figure 7:**
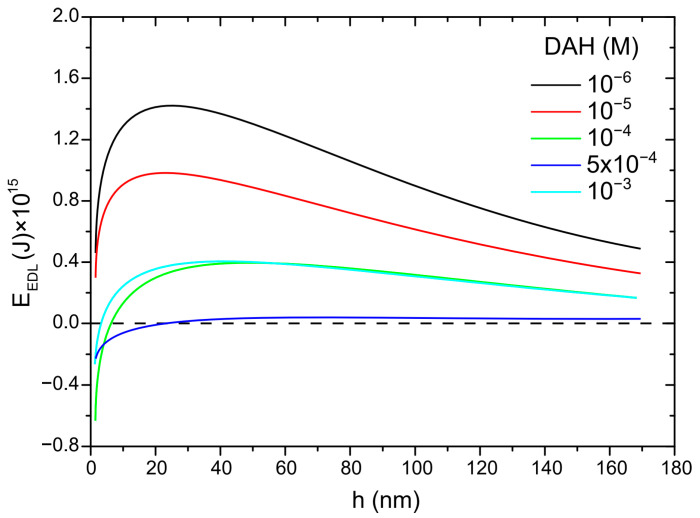
Electrical double-layer energy as a function of DAH concentration (both surfaces are at constant surface potential).

**Figure 8:**
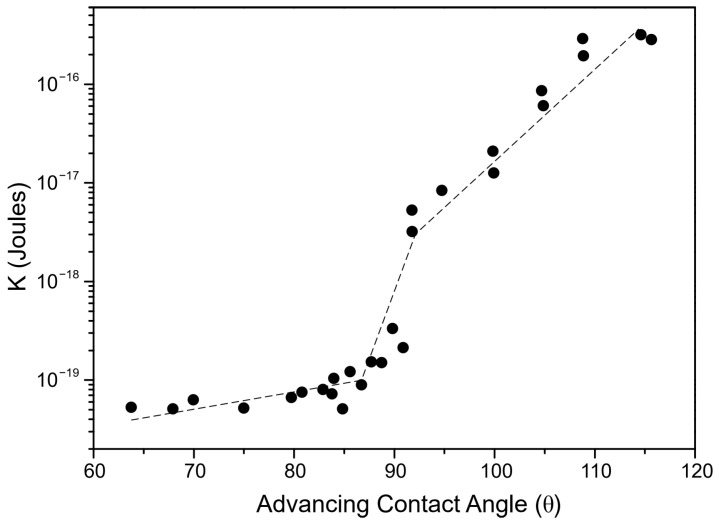
Particle–particle hydrophobic constant vs. advancing contact angle. Adapted from Yoon [2].

**Figure 9:**
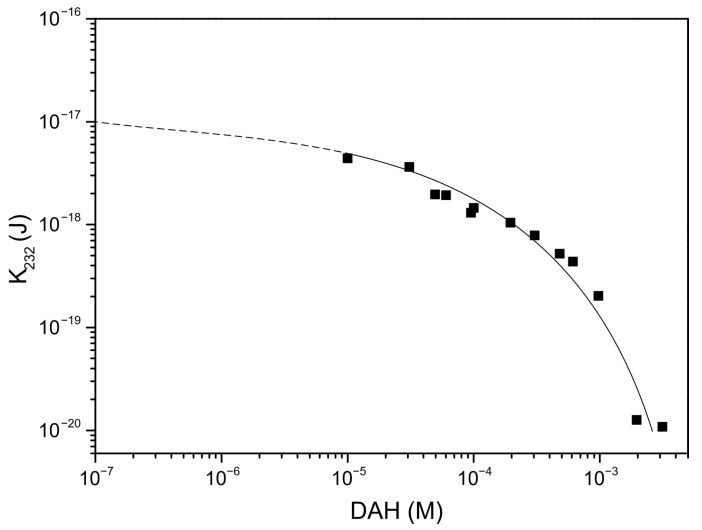
Bubble–bubble hydrophobic constant vs. DAH concentration. Adapted from Yoon and Aksoy [32].

**Figure 10:**
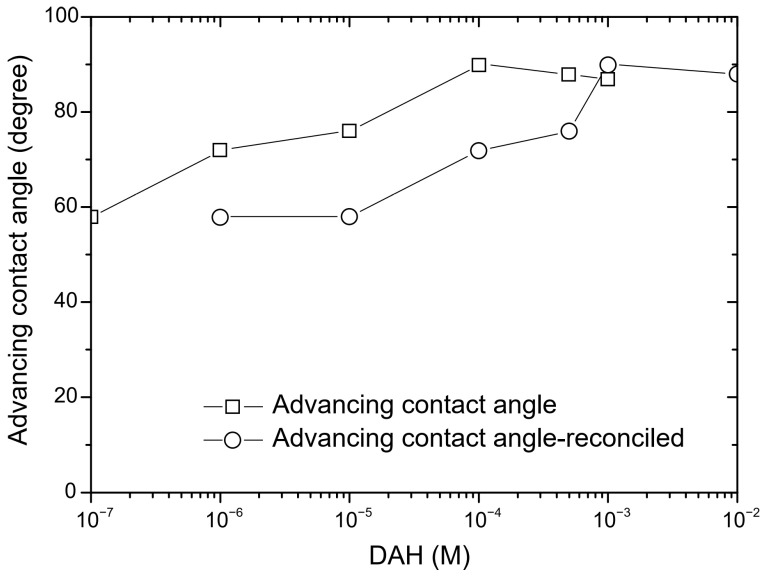
Advancing contact angle as a function of DAH concentration. See text for explanation.

**Figure 11:**
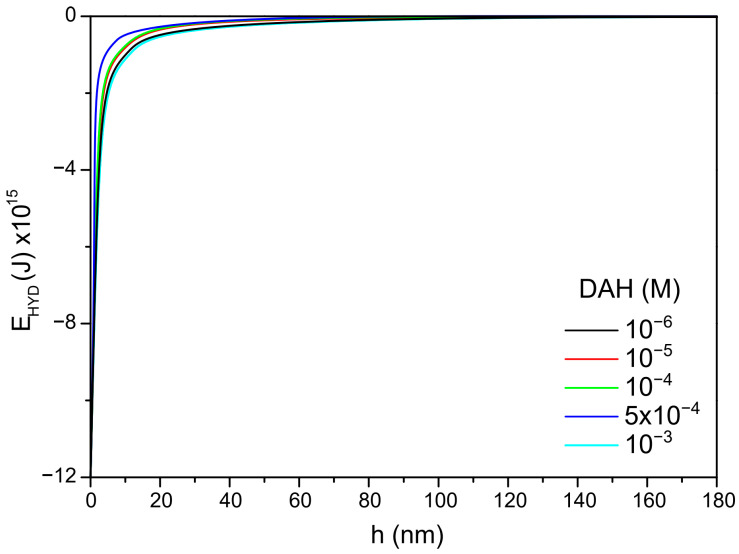
Hydrophobic energy vs. DAH concentration.

**Figure 12:**
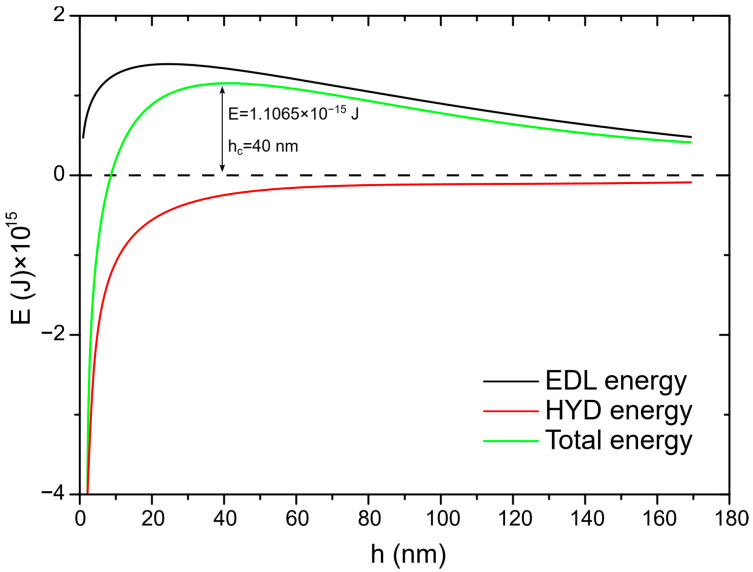
Total energy of bubble–glass bead attachment in the presence of 10^−6^ M DAH. See text for explanation.

**Figure 13:**
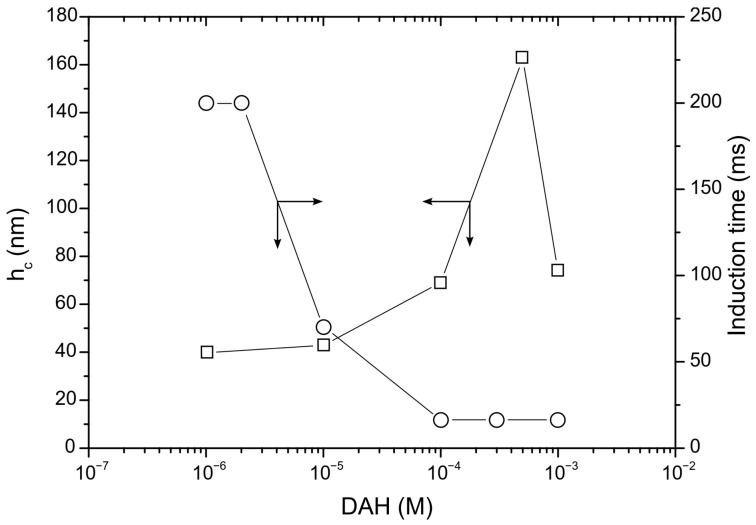
Calculated critical film thickness (squares and left axis) and induction time (circles and right axis) as a function of DAH concentration.

In the next step of calculation, the critical film thickness was determined. This parameter was calculated using the following approach [2]. In this calculation, the overall energy of bubble–particle interactions was determined using the extended DLVO theory, which includes the vdW, the EDL, and the hydrophobic contributions. As mentioned above, the vdW interactions were neglected. To calculate the energy of EDL interactions, zeta potentials were used instead of surface potentials as approximate values. The experimental results for zeta potentials are shown in Figure 6. The results of zeta potential measurements, presented in Figure 7, indicate that the negative zeta potential values for both the particle and bubble decreased with increasing DAH concentration up to 7 × 10^−4^ M. After this concentration, both the particle and the bubble had a positive zeta potential. In other words, the bubble and the particle had the same sign of zeta potential for each DAH concentration, indicating that the energy of EDL interactions was repulsive, except at the DAH concentration where the zeta potential is zero. The energy of EDL interactions was calculated using Equation (7), which assumed that both bubble and particle have constant surface potentials (see Figure 7). While the EDL forces are repulsive in this case, the vdW forces are always repulsive in bubble–particle interactions [3], and the sum of these forces has to be overcome by the so-called attractive hydrophobic force, making the bubble–particle attachment possible. The energy of hydrophobic interactions was calculated using Equation (14). The only unknown parameter, in this equation, was the hydrophobic constant for bubble–particle interactions. To determine this constant, it is important to have values for hydrophobic constants of particle–particle (Figure 8) and bubble–bubble interactions (Figure 9), which are available in Ref. [2]. As seen in Figure 8, it is also important to measure advancing contact angle. In contact angle experiments, atomic force microscopy was used to determine adhesion force, and then Equation (15) was applied to calculate advancing contact angle. It is important to note that in the case of induction time and zeta potential experiments, the solid ratio was 1%, while only one glass particle was used for the advancing contact angle experiments. It means that there is a difference between the adsorption processes of DAH on the glass particle used for contact angle experiments and the glass particles employed in induction time and zeta potential measurements. Because it was found that the maximum contact angle, measured between the polished silica surface and air bubble immersed in different DAH solutions, occurred when the DAH concentration was 10^−3^ M [30], our results for advancing contact angle were reconciled, as seen in Figure 10. Once the advancing contact angle is known, the hydrophobic constant for particle–particle interactions can be obtained. The hydrophobic constant for bubble–particle interactions was calculated as the geometric mean of the hydrophobic constant for particle–particle interactions and the hydrophobic constant for bubble–bubble interactions [2], and then the energy of hydrophobic interactions was determined using Equation (14), as seen in Figure 11. Finally, the total energy of bubble–particle interactions was calculated using Equations (7) and (14), as seen in Figure 12. Based on the maximum of the curve *E_tot_* vs. *h*, the energy barrier for bubble–particle attachment and critical film thickness can be determined. The calculated critical film thicknesses along with induction time measurements are shown in Figure 13. As seen in Figure 13, the critical film thickness is inversely related with induction time. Although we did not experimentally determine critical film thickness, the calculated critical film thickness values are within the range of the values obtained experimentally by Yoon and Yordan [31].

**Figure 14:**
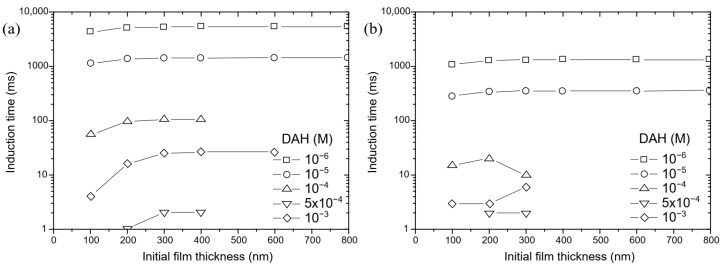
Induction time calculated using the (**a**) Stefan–Reynolds model; and the (**b**) Tsekov et al. 1998 [16] model.

**Figure 15:**
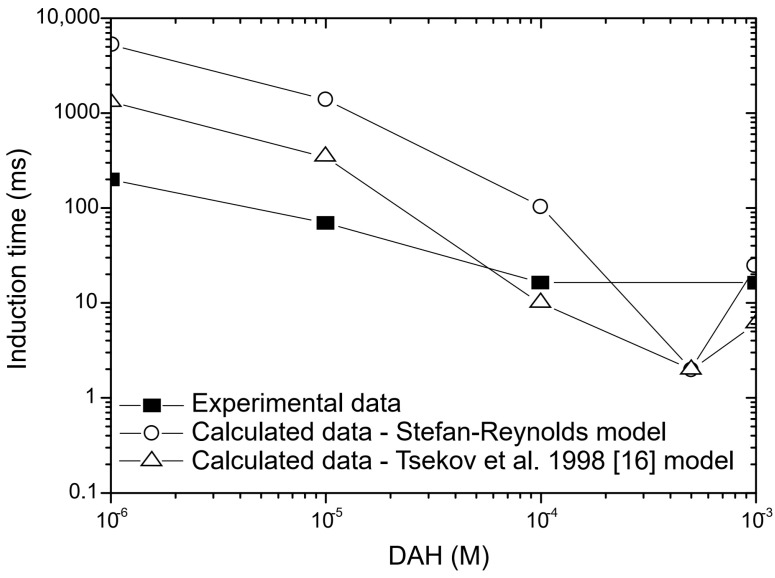
Calculated and experimental induction time as a function of DAH concentration.

Because the mobility of wetting liquid film is well-understood and critical film thickness of wetting films is determined, the induction time can be estimated using the two theoretical models. The first model is the classical Stefan–Reynolds equation, which describes the drainage of liquid film between two plane immobile surfaces. The second model was proposed by Tsekov et al. [16], who extended the Stefan–Reynolds equation by taking into account the surface rheology of adsorbed surfactants. As mentioned above, in our calculation, the complex model of Tsekov et al. [16] is transformed into a simpler model, which can also be derived using the Stefan–Reynolds lubrication approximation with slip boundary conditions. Since the classical Stefan–Reynolds model and the model proposed by Tsekov et al. [16] are differential equations (Equations (1) and (4)) that cannot be solved analytically, the approximation of solutions of these equations was obtained numerically by applying the Runge–Kutta method of the fourth order. The calculated induction time depends on initial film thickness, as shown in Figure 14. Therefore, in this study, the effect of initial thickness of wetting films on calculated induction time was also investigated. By applying either model, the calculated induction time increased with the increase of initial thickness of the wetting film until the initial thickness of the wetting film was 300 nm, and then the calculated induction time had a constant value with further increase of the initial thickness of the wetting film, as shown in Figure 14. Therefore, the initial thickness of the wetting film used in these calculations was 300 nm. Finally, the experimental and calculated induction times as a function of DAH concentration are shown in Figure 15. As seen in Figure 15, at DAH concentration lower than 10^−4^ M, a better agreement between experimental and calculated values determined using the model proposed by Tsekov et al. [16] was achieved, and thus it was confirmed that the mobility of wetting films plays an important role in the drainage of liquid films between a bubble and a particle. When the DAH concentration was 10^−3^ M, the Stefan–Reynolds model gave better prediction than the model proposed by Tsekov et al. [16]. The reason for this is probably because at high DAH concentration, the air–water interface is immobile, and then in this case, the Stefan–Reynolds equation gave a better prediction of induction time than the model proposed by Tsekov et al. [16].

## 4. Materials and Methods

### 4.1. Materials

Acid washed glass beads (150 × 212 μm) purchased from Sigma Aldrich (St. Louis, MO, USA) were used for the induction time measurements. Glass particles (Duke Scientific Corp., Palo Alto, CA, USA) of 15 μm in diameter were used for the AFM particle–bubble force measurements. MilliQ ultrapure water 18.2 MΩ·cm was used in all of the experiments. All glassware was cleaned with alkaline solutions (KOH:H_2_O:Ethanol at 12.5:16:84 mass ratio) followed by vigorously rinsing with ultrapure water. All experiments were conducted at 22 ± 1 °C. Dodecyl amine hydrochloride (97% of C_12_H_24_NH_3_Cl, DAH) obtained from Alfa Aesar (Stoughton, MA, USA) was of reagent grade and used without further purification in all measurements.

### 4.2. Induction Time Experiments

**Figure 16:**
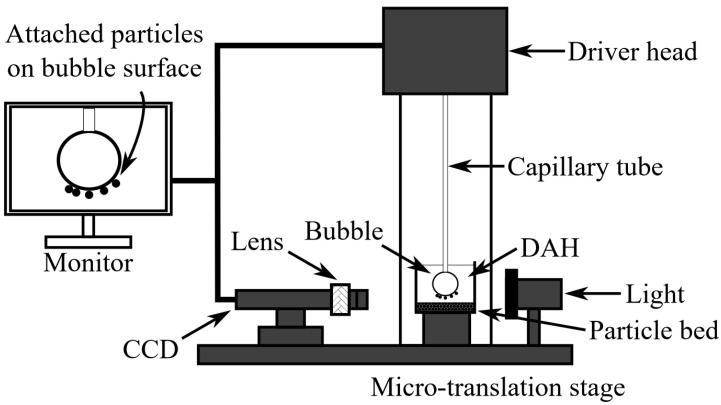
Induction timer setup schematic. Modified from Albijanic et al., 2011 [4].

The induction times for glass particles in the presence of DAH were measured using an Induction Timer (University of Alberta, Edmonton, AB, Canada), with a schematic shown in Figure 16. First, 3 g of glass particles was added to 300 mL of DAH solution, and the suspension was conditioned for 15 min. The conditioned particles and solution were then transferred to a small cell beneath the bubble holder. Induction time experiments were conducted at varying DAH concentrations. During the experiments, a bubble approximately 1.5 mm in diameter was generated using a microsyringe, and the distance between the bubble and the particle bed surface was adjusted using a three-dimensional micro-translation stage. The bubble was then held in contact with the particle bed for a predetermined duration, ranging from 10 to 400 ms. The attachment of glass particles to the bubble was observed visually through a lens and monitored via a CCD camera at a frame rate of 500 frames per second. Ten measurements were conducted at different locations on the upper surface of the particle bed for each contact time. The contact time at which 100% attachment efficiency was observed was recorded as the attachment time.

### 4.3. Zeta Potential Experiments

Zeta potential and particle size measurements for glass beads were conducted as a function of DAH concentration using a Zetasizer Nano-ZS (Malvern, UK). The glass particles were first ground in a laboratory ball mill, then sieved through a 38 µm screen. The undersized fraction was conditioned for 15 min in DAH solutions. Before each measurement, the suspensions were left undisturbed for 5 min to allow coarse particles to settle. A small sample was then extracted from the top of the suspension and transferred to the measurement cell. Five measurements were performed for each DAH concentration, and the average value was calculated, with a measurement error of approximately 4%. Electrophoretic mobility measurements of air bubbles were also conducted using a Microelectrophoresis Apparatus Mk II zeta meter (Rank Brothers, Newbury, UK). The setup and procedures, detailed in a previous study [33], involved dissolving air in 100 mL of deionized water at a constant pressure of 0.5 MPa overnight. A gas-saturated DAH solution was prepared by mixing 9 mL of the gas-saturated water with 1 mL of DAH solution. This mixture was immediately placed in the glass cell of the microelectrophoretic unit, which was then mounted on the cell holder with a pair of electrodes inserted into the cell wings. When a 40 mV external electric field was applied, bubbles moved toward one of the electrodes based on their zeta potential. The mobility of the bubbles, recorded using a CCD camera, was converted to zeta potential using the von Smoluchowski equation [34]. Each experiment was repeated 20 times, and the mean value was used for analysis. The maximum error for these measurements was approximately 6.1%.

### 4.4. Contact Angle Experiments

Contact angle experiments for 15 μm glass particles were conducted as a function of DAH concentration using a bubble–particle force measurement setup with an MFP-3D Atomic Force Microscope (Asylum Research, Santa Barbara, CA, USA). The glass particles were first cleaned using RCA SC-1 solution, as described by Kern and Puotinen [35]. A single glass particle was then attached to the end of a rectangular cantilever (Mikro Masch NSC12/tipless/No Al CA, USA, 0.154 N/m) using a small amount of thermoplastic epoxy resin. For each experiment, a droplet of DAH solution was deposited onto a polytetrafluoroethylene (PTFE) plate using a pipette. An air bubble, approximately 800 μm in diameter, was introduced within the droplet and attached to the PTFE surface by injecting air through a microsyringe. A new bubble was used for each DAH concentration. Approach force curves were measured at a low velocity of 0.5 μm/s to minimize hydrodynamic effects. The advancing contact angle *θ_a_* was calculated using Equation (15) [36]:

(15) $$\theta_{a}=\sqrt{2\arcsin\sqrt{\frac{F_{ad}}{2\pi R\sigma }}}$$

where *R* is the particle radius (15 μm), *F_ad_* represents adhesion force, and *σ* is surface tension.

## 5. Conclusions

This study investigates the influence of intermolecular forces and surface rheology on the drainage of wetting films in the glass beads–DAH system. Measurements of induction time, zeta potential, and contact angle were conducted using an optically-based attachment timer, microelectrophoresis, and atomic force microscopy, respectively. The calculations accounted for the total disjoining pressure and overall energy of bubble–particle interactions, incorporating both DLVO and non-DLVO intermolecular contributions. The critical film thickness was determined based on the overall interaction energy, while the disjoining pressure was utilized to estimate induction time using the classical Stefan–Reynolds equation and the model by Tsekov et al. [16]. The model proposed by Tsekov et al. [16] provided better agreement between experimental and calculated data, highlighting the significant role of surface mobility in the wetting film, driven by the Marangoni effect, in bubble–particle attachment interactions. Additionally, it is crucial to validate the proposed theory by using experimental induction times from real particles with surface roughness and diverse shapes, not only in the presence of DAH, but also with other surfactants under different pulp pH, ionic strengths, and temperatures.

## Data Availability

Data are contained within the article. The raw data supporting the conclusions of this article will be made available by the authors on request.
